# Efficacy analysis of minimally invasive treatment for scapular coracoid fractures assisted by TiRobot ForcePro Superior

**DOI:** 10.3389/fsurg.2025.1639649

**Published:** 2025-08-06

**Authors:** Tan Zhiyun, Dai Yonghong, Li Qingyu, Zeng Yanhui, Yang Kuangyang, Chen Xing

**Affiliations:** ^1^ The Eighth Clinical Medical College of Guangzhou University of Chinese Medicine, Foshan, Guangdong, China; ^2^ Foshan Hospital of Traditional Chinese Medicine, Foshan, Guangdong, China

**Keywords:** minimally invasive, navigation and positioning, scapular coracoid fracture, internal fixation of fracture, robot-assisted surgery lntroduction

## Abstract

**Background:**

Accurate spatial positioning is the key to the precise implantation of coracoid screws. This study aimed to explore the efficacy and current issues of TiRobot ForcePro Superior (TFS) in the treatment of scapular coracoid fractures by comparing it with the freehand screw implantation technique.

**Methods:**

A retrospective analysis was conducted on the medical records of 29 patients with scapular coracoid fractures who underwent surgical treatment at Foshan Hospital of Traditional Chinese Medicine from 2019 to 2024. Based on the surgical approach, the patients were divided into the robot-assisted group (*n* = 15) and the control group (*n* = 14). In the robot-assisted group, the TFS system was utilized to plan the optimal screw trajectory, and the surgeon implanted the guide pin along the mechanical arm sleeve of the TFS, followed by precise screw placement along the guide pin to fix the coracoid fracture. In the control group, screws were implanted freehand by the surgeon.

**Results:**

The intraoperative blood loss and incision length in the robot-assisted group were significantly less than those in the control group. The visual analog scale (VAS) pain scores in the robot-assisted group were significantly lower than those in the control group. The shoulder function scores in the robot-assisted group were significantly higher than those in the control group. No statistically significant differences were observed between the two groups in terms of operative time, hospital stay, screw placement accuracy, incidence of postoperative complications, or fracture healing time.

**Conclusion:**

Compared with freehand screw implantation, minimally invasive treatment for scapular coracoid fractures assisted by TFS significantly reduced intraoperative blood loss, shortened incision length, alleviated pain, and better promoted the recovery of shoulder joint function.

## Introduction

Scapular fractures account for approximately 0.4% to 1% of all fractures, while coracoid fractures of the scapula are relatively rare, constituting only 2% to 5% of scapular fractures. Their occurrence is predominantly associated with high-energy direct trauma (1–3). The coracoid process has a unique anatomical location and an irregular shape. The screw pathway at the junction of the coracoid and the scapular neck is narrow, and the surrounding area is densely populated with nerves and blood vessels. Traditional freehand screw placement heavily relies on the surgeon's experience, posing significant risks and challenges. With the increasing application of robotic technology in clinical practice, new approaches for minimally invasive treatment of coracoid fractures have emerged. However, research on the efficacy of robot-assisted minimally invasive treatment for coracoid fractures is limited, with most studies being case reports (4).The TiRobot ForcePro Superior is a navigation and positioning robot capable of achieving submillimeter spatial localization (5–9). Currently, there is no research available on the application of TiRobot ForcePro Superior in the treatment of coracoid fractures. This study aims to compare the efficacy of robot-assisted screw placement with traditional freehand screw placement, thereby further exploring the effectiveness of robot-assisted minimally invasive treatment for coracoid fractures and providing a reference for clinical management of such fractures.

## Methods

### Inclusion and exclusion criteria

The inclusion criteria were as follows: (1) imaging examination revealing significant displacement in Eyres type I to V fractures; (2) isolated coracoid fractures or combined injuries involving other structures of the superior shoulder suspensory complex (SSSC); (3) active patients who required normal shoulder function and desired a swift return to work or daily habits. The exclusion criteria were: (1) concurrent ipsilateral upper limb fractures or dislocations; (2) a history of shoulder diseases with severely compromised function; (3) severe underlying conditions rendering the patient unfit for anesthesia and surgery; (4) pathological fractures; (5) inability to effectively fix the tracer; (6) active infection in or near the surgical area.

After applying the aforementioned inclusion and exclusion criteria, a total of 29 patients with coracoid fractures were enrolled and divided into the robot-assisted group and the control group based on the method of coracoid screw placement. The baseline characteristics of the two groups showed no statistically significant differences (*P* > 0.05), indicating comparability, as shown in Table 1.

**Table 1:** Comparison of baseline data between the two groups.

Baseline data	Robot-assisted group (*n* = 15)	Control group (*n* = 14)	Statistical value	*P*-value
Gender(Male/Female), n	11/4	9/5	—	0.700
Age, years	47.20 ± 11.53	44.29 ± 12.92	*t* = 0.642	0.526
BMI, kg/m^2^	23.58 ± 3.53	23.31 ± 3.54	*t* = 0.209	0.836
Injury mechanism, n			*χ*^2^ = 1.727	0.715
Accidental falls	3	3		
Fall from height	5	2		
Traffic accident	5	7		
Crush injury caused by heavy objects	2	2		
Injury-to-surgery time, d	10（6, 12）	10（7.75, 13.5）	*Z* = −0.110	0.913
Type of injury			—	0.413
Injury involving ≥2 sites of the SSSC, n	8	9		
Isolated coracoid process fracture, n	7	5		
Eyres classification of fractures(ⅠA/ⅠB/ⅡA/ⅡB/ⅢA/ⅢB/ⅣA/ⅣB/ⅤA/ⅤB), n	0/0/0/2/2/1/3/1/1/5	0/2/1/0/0/0/1/3/3/4	χ^2^ = 9.671	0.249
Follow-up duration, months	13（11, 23）	19.5（12.75, 21.5）	*Z* *=* −0.791	0.429

SSSC denotes the superior shoulder suspensory complex; “—” indicates no statistical value was obtained using Fisher's exact test.

### Introduction to TiRobot ForcePro Superior

**Figure 1:**
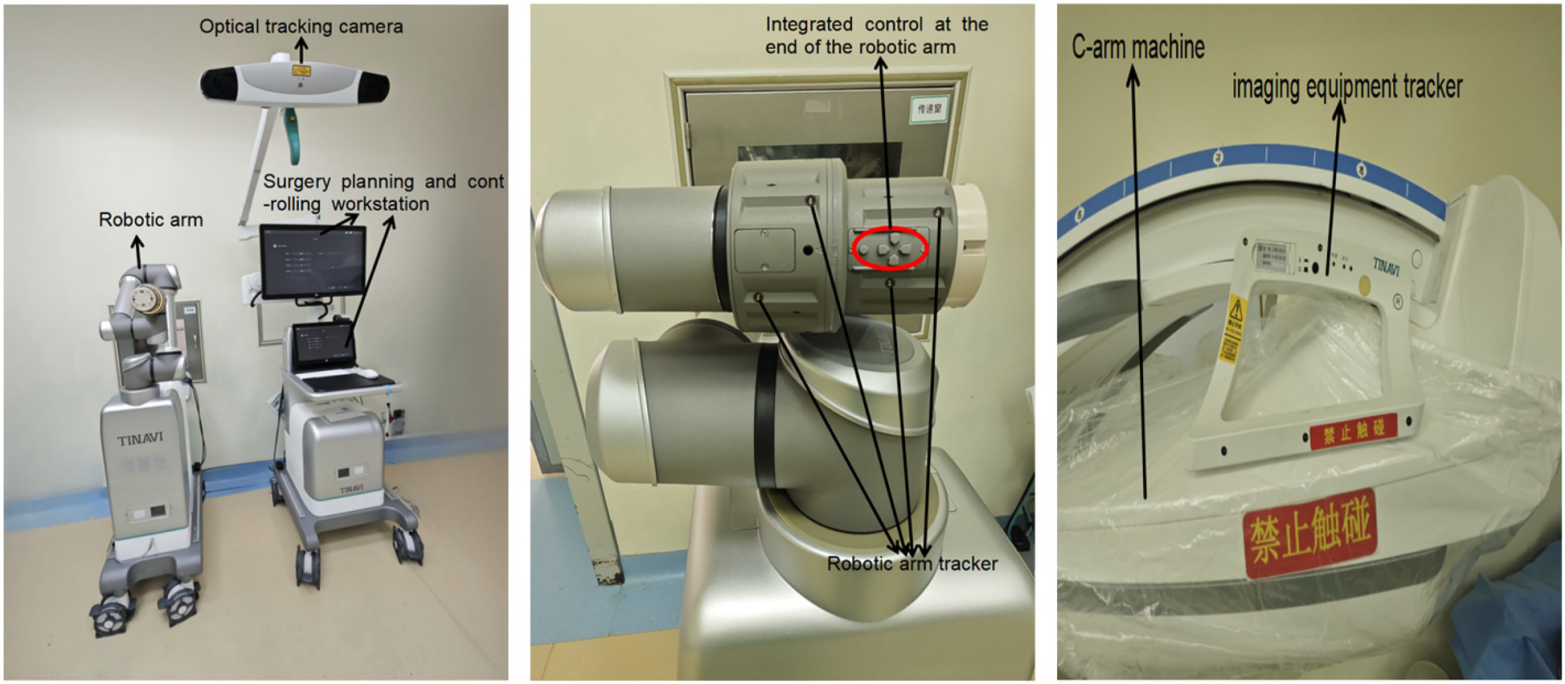
Components of TiRobot ForcePro Superior.

TiRobot ForcePro Superior was developed by Beijing TINAVI Medical Technologies Co., Ltd., China. TFS primarily consists of a robotic arm, a surgery planning and controlling workstation, an optical tracking camera, and an imaging equipment tracker (Figure 1).

### Surgical procedure

**Figure 2:**
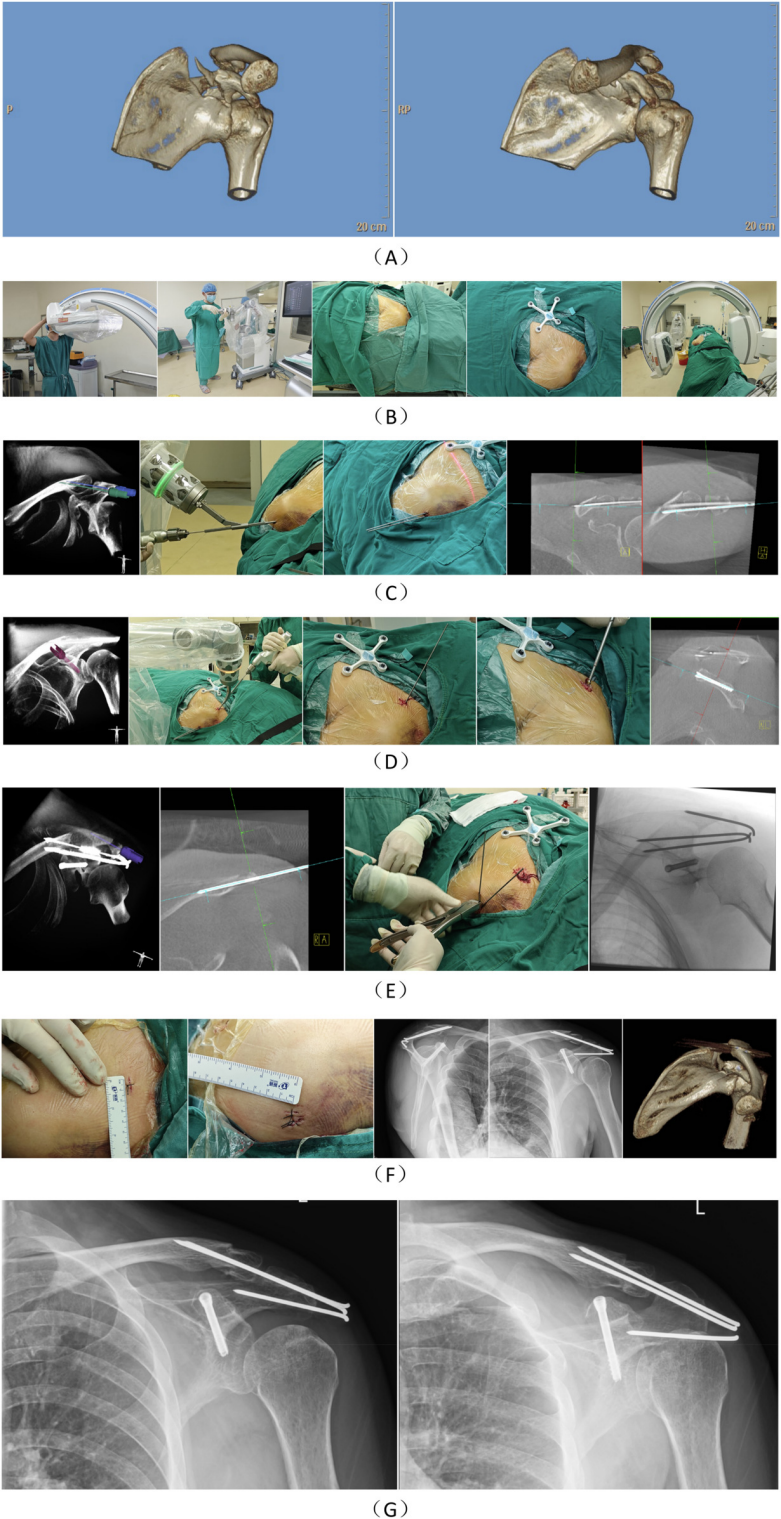
Case presentation: A 54-year-old male patient sustained a fracture of the left scapular coracoid process due to an accidental fall, accompanied by a left clavicle fracture, fractures of the left 2nd to 8th ribs, and pulmonary contusion. **(A)** The fracture morphology of the patient was characterized by a coracoid base fracture extending into the scapula. **(B)** A sterile working environment was established for the robotic arm and the image intensifier of the C-arm x-ray machine using a disposable sterile protective cover. After routine disinfection and draping, the patient tracker was installed. Three-dimensional imaging data of the surgical area were collected and uploaded to the robotic surgical planning platform. **(C)** The surgical paths for two Kirschner wires were first planned, and the Kirschner wires were implanted with robotic assistance. The primary objective of this step was to initially reduce and fix the clavicle to prevent the coracoclavicular ligament from interfering with the reduction of the coracoid fracture. **(D)** The coracoid screw was planned, and a guide wire was implanted with robotic assistance, followed by the insertion of the screw along the guide wire. **(E)** Another Kirschner wire was planned to fix the acromion. After completing all internal fixations of the fractures, the Kirschner wires were bent and cut. **(F)** The patient's postoperative incision condition and postoperative imaging were documented. **(G)** The patient's x-ray images at 4 months postoperatively showed that the fracture had healed.

a. General anesthesia was administered, and the patient was positioned supine on a radiolucent operating table with the affected shoulder elevated and the head tilted to the contralateral side. The affected upper limb was flexed and secured to the body.
b. The surgical area was routinely disinfected and draped.
c. The TiRobot ForcePro Superior was powered on, and the robotic arm was deployed. Both the robotic arm and the C-arm x-ray machine (Siemens, Germany) were covered with sterile protective sleeves to establish an aseptic working environment.
d. The console was positioned at the caudal end, with the optical camera directed toward the center of the surgical field.
e. The robotic arm was placed on the affected side at an angle of 45°–90° relative to the head of the operating table.
f. A patient tracker was fixed to the ipsilateral acromion or proximal clavicle or attached to the chest wall, oriented caudally. The C-arm x-ray machine was positioned on the contralateral side of the robotic arm, and three-dimensional imaging data of the coracoid process were acquired for automatic image registration.
g. Following image registration, the entry point, exit point, diameter, and length of the screw were planned on the robotic intelligent surgical planning platform.
h. After planning, a robotic arm motion simulation was conducted to confirm safe accessibility to the target position. Upon completion of the simulation, a guide pin was inserted with robotic assistance. After verifying satisfactory guide pin placement, a cannulated lag screw was inserted along the guide pin. The screw position was confirmed as satisfactory, and the guide pin was subsequently removed.
i. For patients with concurrent acromion fractures, distal clavicle fractures, or acromioclavicular joint dislocations, manual reduction was performed first, followed by internal fixation using Kirschner wires under TFS assistance to facilitate subsequent coracoid fracture reduction.
j. The incision was sutured, and the procedure was concluded. A representative case is shown in Figure 2.

### Postoperative management

Routine anti-infection, anti-swelling, and analgesic treatments were administered postoperatively. x-ray imaging was reviewed on the second postoperative day. Passive functional exercises were initiated on the fourth postoperative day. If only acromioclavicular joint dislocation was present, active functional exercises were started in the third postoperative week. When one or more fractures coexisted in addition to the coracoid fracture, shoulder joint mobility was limited to 90° within the first three weeks postoperatively, and resistance exercises were initiated in the sixth postoperative week, with shoulder joint mobility exceeding 90°. Patients were prohibited from carrying heavy objects for two months postoperatively.

### Observation metrics

The incision length, operative time, and intraoperative blood loss of the patient were recorded during the surgery. The excellent and good rate of screw placement was documented based on postoperative three-dimensional CT reconstruction. The qualitative evaluation criteria for screw placement were as follows (10): excellent, the screw was entirely within the bone channel without cortical violation; good, the screw position was safe, with partial contact to the cortical bone but no penetration; poor, the screw penetrated the cortical bone or entered the joint. The length of hospital stay was recorded at discharge. During follow-up after discharge, x-ray imaging was reviewed, and the healing time of the coracoid fracture and the occurrence of postoperative complications were documented. At the final follow-up, the Visual Analog Scale (VAS) for pain and the Constant-Murley Score (CMS) (11) were recorded to evaluate the recovery of shoulder joint function.The Constant-Murley Score for all patients was evaluated by the same observer.

### Statistical analysis

Statistical analysis was performed using IBM SPSS Statistics 26. Categorical data were expressed as counts and percentages [*n* (%)], and comparisons between groups were conducted using Fisher's exact test. Normally distributed continuous data were expressed as mean ± standard deviation. For normally distributed data with homogeneity of variance, independent samples *t*-test was used for between-group comparisons; in cases of heterogeneity of variance, the corrected *t*-test (t' test) was applied. Non-normally distributed continuous data were expressed as median (lower quartile, upper quartile), and between-group comparisons were performed using the non-parametric rank-sum test. A *P*-value < 0.05 (two-tailed test) was considered statistically significant.

## Results

The intraoperative blood loss and incision length in the robot-assisted group were significantly lower than those in the control group, with the differences being statistically significant (*P* < 0.05). No statistically significant differences were observed between the two groups in terms of operation time and hospital stay (*P* > 0.05).

Regarding the accuracy of screw placement, the excellent and good rate of screw positioning in the TiRobot group was 95.24%, compared to 66.67% in the control group. However, due to the small sample size, the difference between the two groups was not statistically significant.

There was no statistically significant difference in fracture healing time between the two groups (*P* > 0.05).

At the final follow-up, the Visual Analogue Score (VAS) in the robot-assisted group was significantly lower than that in the control group, while the Constant-Murley Score (CMS) was significantly higher, with the differences being statistically significant (*P* < 0.05).

Regarding postoperative complications, in the control group, one patient with Eyres type IVB fracture experienced screw displacement, one patient with type VB fracture developed malunion, and one patient each with type IB, IVB, VA, and VB fractures exhibited screw penetration through the cortical bone. In the robot-assisted group, one patient with type VA fracture experienced screw penetration through the cortical bone. However, the difference in postoperative complications between the two groups was not statistically significant (*P* > 0.05). A comparison of outcome measures between the two groups is presented in Table 2.

**Table 2:** Comparison of outcome measures between the two groups.

Outcome indicators	Robot-assisted group (*n* = 15)	Control group (*n* = 14)	Statistical value	Effect value (95%*CI*)	*P-*value
Operation time, min	120（85, 140）	114（89, 162.75）	*Z* = −0.131	*MD* = −0.5（−43, 33）	0.896
Blood loss, ml	20（10, 50）	200（170, 325）	*Z* = −3.704	*MD* = −185（−195, −70）	0.000
Incision length, cm	1（1, 2）	10（5.75, 12）	*Z* = −3.844	*MD* = −6（−9, −4）	0.000
Hospital stay, d	15（13, 15）	14.5（10.75, 20.25）	*Z* = −0.220	*MD* = 0（−4, 3）	0.826
Screw positions, n			—	—	0.083
Excellent	18	9			
Good	2	1			
Poor	1	5			
Fracture healing time, months	2.74 ± 0.39	2.96 ± 0.49	* t* = −1.374	*MD* = −0.22（−0.56, 0.11）	0.181
Visual Analogue Scale, points	0（0, 0）	1（0, 3）	* Z* = −2.382	*MD* = −1（−2, 0）	0.017
Constant-Murley Score, points	100（99, 100）	85.50（78.75, 90）	*Z* = −3.822	*MD* = 14（10, 20）	0.000
Postoperative complications, *n* (%)					
Screw penetration through the cortical bone	1 (6.67)	4 (28.57)	—	*OR*=0.18（0.02, 1.85）	0.169
Screw loosening and displacement	0 (0)	1 (7.14)	—	*OR*=1.08（0.93, 1.25）	0.483
Malunion of fracture	0 (0)	1 (7.14)	—	*OR*=1.08（0.93, 1.25）	0.483

## Discussion

The SSSC (Superior Shoulder Suspensory Complex) is a bony-ligamentous ring structure composed of the superior glenoid, coracoid process, coracoclavicular ligament, coracoacromial ligament, lateral end of the clavicle, acromioclavicular joint, and acromion. The coracoid process serves as the attachment site for the pectoralis minor, coracobrachialis, and short head of the biceps brachii muscles, as well as the origin of the coracoacromial ligament, coracohumeral ligament, coracoclavicular ligament, and superior transverse scapular ligament. It is a critical structure for the integrity of the SSSC, the “coracoacromial arch,” and the anterior-superior stability of the shoulder joint. Currently, there is no consensus on the treatment of coracoid fractures. Eyres suggests that Eyres types I to III fractures, regardless of displacement, can be managed conservatively, while surgical intervention is recommended for Eyres types IV to V fractures, Eyres type III fractures associated with acromioclavicular joint dislocation, or cases where the coracoid fracture fragment obstructs shoulder joint reduction (12). Anavian et al. (13)propose that the surgical indications for coracoid fractures include: (1) fracture displacement greater than 1 cm; (2) multiple disruptions of the SSSC; (3) coracoid fractures combined with scapular fractures requiring surgical treatment; and (4) failure of conservative management.

The fracture lines of Eyres types I to II primarily involve the tip and body of the coracoid process. The coracoid tip serves as the attachment site for the coracoacromial ligament, the short head of the biceps brachii, and the conjoint tendon of the coracobrachialis muscle. The coracoid body is the attachment site for the coracoacromial ligament, coracoclavicular ligament, and the tendon of the pectoralis minor muscle. As the tip and body are critical attachment points for the ligaments and muscles of the SSSC (Superior Shoulder Suspensory Complex) and the “coracoacromial arch,” significant fracture displacement can compromise shoulder joint stability. Additionally, the lack of compression fixation at the fracture site may lead to further displacement during upper limb exercises, thereby affecting fracture healing. Furthermore, conservative treatment prolongs the immobilization period of the upper limb, potentially causing shoulder joint stiffness and requiring extended rehabilitation to restore normal upper limb function for daily activities. Therefore, the author believes that early surgical intervention can provide a more stable bony attachment site for the muscles and ligaments around the coracoid process. Moreover, robot-assisted surgery is minimally invasive, facilitating early functional exercise and rehabilitation, and reducing the overall duration of the condition.

### Analysis of the efficacy of TiRobot ForcePro Superior-assisted minimally invasive treatment for scapular coracoid fractures

In terms of surgical duration, the robot-assisted group was slightly longer than the control group. This was attributed to the low incidence of coracoid fractures and the limited number of patients willing to undergo the higher-cost robotic surgery, resulting in fewer opportunities for the surgical team to operate the robot for coracoid fractures. Consequently, the initial proficiency level was lower, leading to longer surgical times. However, the overall difference in surgical duration was not statistically significant. With accumulated experience, the shortest surgical time in the robot-assisted group was 47 min, achieving or even surpassing that of the control group.

In terms of blood loss and incision length, it was considered that the control group involved the surgical management of multiple injuries simultaneously. Therefore, the incision length and blood loss from other shoulder injuries treated following the robotic surgery were also included to enhance comparability between the two groups. Even so, the blood loss and incision length in the robot-assisted group were significantly smaller than those in the control group, with the difference being statistically significant. This demonstrated that robot-assisted minimally invasive treatment of coracoid fractures effectively reduced trauma, which facilitated rapid postoperative recovery for patients. The structures surrounding the coracoid process are complex, with the suprascapular nerve passing above the base and the brachial plexus and axillary vessels running medially. Inaccurate intraoperative positioning could easily lead to vascular or nerve injuries. The TiRobot ForcePro Superior system incorporates intelligent algorithms for screw pathway calculation, enabling precise spatial positioning and reducing the risk of iatrogenic neurovascular injuries.

A study by Yoshiteru Kawasaki et al. (14) demonstrated that the minimum short-axis diameter at the narrowest part of the coracoid base was 6.6 mm, making it difficult for screws to pass through the coracoid base fracture site into the scapular neck. In terms of screw placement accuracy, the excellent and good rate of screw positioning in the robot-assisted group was 95.24%, significantly higher than the 66.67% in the control group. However, due to the small sample size, the difference was not statistically significant. In the robot-assisted group, one patient had multiple fractures of the acromion and clavicle, resulting in unstable temporary fixation with Kirschner wires. This led to slight relative displacement between the patient tracker and the surgical site. Additionally, the TiRobot ForcePro Superior (TFS) theoretically has a precision error of approximately 1 mm, Combined with the narrow screw pathway at the coracoid base, this error was further amplified, leading to one case of screw penetration through the cortical bone in the robot-assisted group. Since the penetration was minimal, no pain or functional impairment was observed during follow-up. During manual guide pin placement, the irregular anatomical morphology of the coracoid and the unavoidable physiological tremor of the surgeon's hands made it difficult to precisely control the direction of the guide pin, resulting in significant operational errors and challenges in accurately placing the screw in the ideal position. Manual guide pin placement requires a high level of spatial awareness and operational experience from the surgeon. However, after planning the screw entry point, exit point, length, and diameter on the TFS surgical planning platform, the sleeve mounted on the robotic arm could determine the trajectory of the guide pin. Using the planned screw length as the limit for the depth of the guide pin after entering the bone, the surgeon could safely and accurately place the guide pin into the target position, providing a completely rigid pathway for screw placement. Theoretically, dual-screw fixation offers better anti-rotational stability compared to single-screw fixation, but the surgical procedure for placing two screws is more challenging than that for a single screw. In the robot-assisted group, six patients achieved dual-screw fixation, while only one patient in the control group achieved dual-screw fixation. The TFS system can plan multiple screw pathways at once, with the robot autonomously positioning them sequentially, simplifying the surgical procedure while further enhancing the stability of the fracture site.

In terms of fracture healing, there was no statistically significant difference in the healing time between the two groups. However, one case of malunion was observed in the control group in a patient with Eyres type VB fracture. Based on postoperative CT scans, the malunion was attributed to inadequate reduction. In Eyres type V fractures, the coracoacromial ligament, coracohumeral ligament, and pectoralis minor are located laterally, while the coracoclavicular ligament is attached to the bone fragment, making direct visualization of reduction intraoperatively impossible. Due to the unique anatomical position of the coracoid process, intraoperative fluoroscopy was challenging. When obtaining anteroposterior shoulder radiographs, the images were often obscured by the humeral head and scapula. The patient's positioning also made it difficult to obtain scapular Y-view and Stryker notch radiographs, and changes in shoulder joint position could further exacerbate fracture displacement. Surgeons typically assessed reduction by placing their fingertips at the base of the coracoid process and palpating the fracture line, making it difficult to accurately judge the quality of fracture reduction. In Eyres type V fractures, the fracture line primarily involved the anterosuperior glenoid articular surface. Since the most critical articular surface was not affected, even non-anatomical reduction had minimal impact on shoulder joint function (15).The robot was capable of performing surgical procedures within confined spaces, causing minimal disturbance to the surrounding soft tissues, which theoretically should have resulted in shorter fracture healing times. However, due to the limited sample size, this difference was not statistically significant.

In terms of pain relief and shoulder joint functional recovery, the robot-assisted group outperformed the control group. This was attributed to the deep anatomical location of the coracoid process, which is surrounded by abundant muscle coverage and adjacent to critical neurovascular structures. Traditional surgical approaches were associated with greater trauma, increased blood loss, and a higher risk of iatrogenic injuries. Robot-assisted surgery, characterized by precision and safety, allowed for the planning of completely osseous screw pathways that effectively avoided important neurovascular structures, minimizing surgical trauma. Consequently, patients experienced faster pain relief and were able to engage in early postoperative functional activities, promoting functional recovery. Additionally, the smaller skin incisions and reduced soft tissue damage lowered the risk of infection. However, one patient in the robot-assisted group had a lower Constant-Murley (CM) score. This patient presented with preoperative suprascapular nerve injury, supraspinatus tendon injury, and subscapularis tendon injury, resulting in poor postoperative rehabilitation outcomes. During follow-up, the patient still exhibited limited upper limb abduction and shoulder pain. Since the nerve injury occurred preoperatively, it was not classified as a postoperative complication in the robot-assisted group.

In terms of postoperative complication rates, the complication rate in the robot-assisted group was 6.67% (1/15), while that in the control group was 42.86% (6/14). This demonstrated that the application of robot-assisted surgery could control the error in screw placement angle and distance to within millimeters, significantly reducing complications. However, due to the small sample size, the difference was not statistically significant. In this study, one case of screw loosening and displacement occurred in a type IVB patient in the control group, while no such cases were observed in the robot-assisted group. This was attributed to the robot's ability to plan screw placement in areas with greater bone density before implantation, effectively preventing screw loosening and displacement.

### Precautions for TiRobot ForcePro Superior-assisted minimally invasive treatment of scapular coracoid fractures

When utilizing TFS-assisted minimally invasive treatment for coracoid fractures, the following points should be noted: (1) The distance between the coracoid process and surrounding neurovascular structures varies with changes in patient positioning and arm position; the coracoid is farthest from neurovascular structures when the patient is in the supine position (16, 17). (2) Factors such as suboptimal image acquisition, deviations in tool kit precision, excessive entry angles causing slippage, loosening of patient trackers, or mismatched tool kits or instruments can affect the accuracy of robotic surgery and should be carefully monitored and prevented. (3) Due to the abundance of neurovascular structures around the coracoid process and the low margin for error, the robotic arm sleeve must be firmly pressed against the bone surface during guide pin insertion to prevent guide pin slippage. (4) Coracoid fractures are often accompanied by other shoulder injuries such as acromioclavicular joint dislocation or clavicle fractures. When using TFS-assisted screw placement, temporary reduction and fixation of the acromioclavicular joint and clavicle with Kirschner wires should be performed first to reduce the traction of the coracoclavicular ligament on the coracoid, facilitating fracture reduction. (5) If any change in fracture position is detected, three-dimensional imaging data of the surgical area should be reacquired, and the surgical path should be replanned to avoid surgical errors.

### Limitations of TiRobot ForcePro Superior

TFS has several limitations: (1) TFS employs optical tracking technology, which is susceptible to obstruction by objects in the operating room space; (2) TFS cannot display the depth of guide pin insertion in real time, necessitating fluoroscopy to verify the depth of guide pin penetration into the bone; (3) TFS cannot provide real-time visualization of fracture reduction status; (4) Since intraoperative maneuvers by the surgeon may alter fracture position, TFS cannot detect such changes and thus cannot actively alert the surgeon.

Additionally, this study has several limitations. Firstly, it is a single-center study; therefore, future research should involve multiple centers in collaboration with other hospitals to further validate the findings. Secondly, due to the low incidence of coracoid fractures, the number of cases included in this study is relatively small, and future studies should aim to increase the sample size. Lastly, this study is retrospective, and prospective validation studies should be conducted when conditions permit in the future.

## Conclusion

In summary, compared to traditional open surgery, robotic surgery offers advantages such as greater precision, smaller incisions, reduced trauma, less bleeding, faster pain relief, better functional recovery, and fewer postoperative complications. Additionally, the strong operability of robotic technology significantly reduces the learning curve for fracture surgeries involving complex anatomical relationships and irregular bone morphology.

## Data Availability

The original contributions presented in the study are included in the article/Supplementary Material, further inquiries can be directed to the corresponding author.
